# Women's Choice to Deliver at Home: Understanding the Psychosocial and Cultural Factors Influencing Birthing Choices for Unskilled Home Delivery among Women in Southwestern Uganda

**DOI:** 10.1155/2020/6596394

**Published:** 2020-06-03

**Authors:** Esther C. Atukunda, Godfrey R. Mugyenyi, Celestino Obua, Angella Musiimenta, Edgar Agaba, Josephine N. Najjuma, Norma C. Ware, Lynn T. Matthews

**Affiliations:** ^1^Mbarara University of Science and Technology, Mbarara, Uganda; ^2^Global Health and Social Medicine, Harvard Medical School, MA, USA; ^3^Massachusetts General Hospital, Division of Infectious Diseases and Center for Global Health, Boston, Massachusetts, USA; ^4^University of Alabama at Birmingham, Division of Infectious Disease, Birmingham, AL, USA

## Abstract

**Background:**

Utilization of perinatal services in Uganda remains low, with correspondingly high rates of unskilled home deliveries, which can be life-threatening. We explored psychosocial and cultural factors influencing birthing choices for unskilled home delivery among postpartum women in rural southwestern Uganda.

**Methods:**

We conducted in-depth qualitative face-to-face interviews with 30 purposively selected women between December 2018 and March 2019 to include adult women who delivered from their homes and health facility within the past three months. Women were recruited from 10 villages within 20 km from a referral hospital. Using the constructs of the Health Utilization Model (HUM), interview topics were developed. Interviews were conducted and digitally recorded in a private setting by a native speaker to elicit choices and experiences during pregnancy and childbirth. Translated transcripts were generated and coded. Coded data were iteratively reviewed and sorted to derive categories using inductive content analytic approach.

**Results:**

Eighteen women (60%) preferred to deliver from home. Women's referent birth location was largely intentional. Overall, the data suggest women choose home delivery (1) because of their financial dependency and expectation for a “*natural*” and normal childbirth, affecting their ability and need to seek skilled facility delivery; (2) as a means of controlling their own birth processes; (3) out of dissatisfaction with facility-based care; (4) out of strong belief in fate regarding birth outcomes; (5) because they have access to alternative sources of birthing help within their communities, perceived as “*affordable*,” “*supportive*,” and “*convenient*”; and (6) as a result of existing gender and traditional norms that limit their ability and freedom to make family or health decisions as women.

**Conclusion:**

Women's psychosocial and cultural understandings of pregnancy and child birth, their established traditions, birth expectations, and perceptions of control, need, and quality of maternity care at a particular birthing location influenced their past and future decisions to pursue home delivery. Interventions to address barriers to healthcare utilization through a multipronged approach could help to debunk misconceptions, increase perceived need, and motivate women to seek facility delivery.

## 1. Introduction

An estimated 300,000 women die each year from preventable causes related to pregnancy and childbirth: approximately 95% occur in developing countries [1, 2]. Almost two decades ago, low maternity service utilization and high maternal deaths in Uganda were attributed to lack of skilled staff at health facilities, poor or inadequate maternity care services, and community's adherence to traditional birthing practices and beliefs that pregnancy was a test of one's endurance and that maternal death was a sad but normal event [3]. With improved community education and engagement over the years, plus increased skilled capacity at health facilities, perinatal service utilization in Uganda remains unacceptably low with correspondingly high rates of unskilled home deliveries, which can be life-threatening [2, 4, 5]. Uganda has one of the highest maternal (>360 for every 100,000 women) and perinatal (41 deaths per 1000 births) mortality ratios in the world [6].

Uganda's public health system is organized in seven tiers with national and regional referral hospitals, general district hospitals, and four levels of health centers (HCs) at community (HC1), parish (HC2), subcounty (HC3), and county (HC4) levels. Staffing and available services vary across the four levels: HC3 and HC4 should offer Emergency Obstetrics Care (EMOC), whereas HC1 and HC2 serve as low resource referral units which are not able to provide EMOC and have no ambulances and blood transfusion services [7, 8]. CSOs and private providers operate in parallel to the public system, and in the case of maternal health, the services of CSOs often cut across several activities at various levels of health care provision [9]. With the increasing availability of maternity services, over 90% of women attend at least one ANC visit, and less than 60% attend at least 4 ANC visits, of the 8 recommended by the WHO [6, 10]. Only 70% of women deliver in a health facility or in the presence of a skilled health personnel, depicting a high disparity in maternity service utilization despite the government's effort to improve accessibility to these services.

Many beliefs and misconceptions about pregnancy and childbirth may influence women's birth choices, which in turn affects health outcomes of both the mother and the baby [11], with risks increasing in lower resourced settings, where birth preparedness and/or infection prevention is not paramount. A person's intentions to engage in a specific behavior (e.g., not attending a scheduled ANC visit, delivering at home) can also be determined by her attitude towards the behavior, her perceived ability (control) to perform a behavior (e.g., to manage her pregnancy or deliver by self at home), and the prevailing normative and subjective beliefs regarding that behavior (e.g., about pregnancy and child birth) within her larger cultural or social context [12, 13]. The individual's intention or motivation to perform or execute a certain behavior (e.g., deliver at home) can further be facilitated by the presence of enabling factors and/or enabling environment (perceived power) within which they live, and such intentions (over time) “will predict the likelihood of that individual actually engaging in that given behavior at some point in the future” [12, 14].

To avert maternal and perinatal deaths, the WHO has called for the development and evaluation of adaptable and context-specific health solutions to promote ANC uptake and empower women to overcome barriers to care and facility delivery [15]. Identifying and scaling up such interventions to improve access to quality healthcare in pregnancy and childbirth have great potential to prevent a substantial number of the annual 823,000 stillbirths, 1,145,000 neonatal deaths, and 166,000 maternal deaths in the 75 highest burden countries [16–19]. Despite some successes in pilot studies and enthusiasm from the public sector, interventions to motivate improved outcomes among pregnant women often neglect to base interventions in behavioral theory and have therefore not been brought to scale [20–22]. According to several scholars, motivating health care utilization is a key element of developing effective behavior change interventions [3, 23–28]. Understanding psychosocial and cultural factors that motivate women to choose and/or participate in unskilled home delivery is needed to inform theory or interventions targeting women in rural southwestern Uganda.

## 2. Methods

### 2.1. Study Design

This study used a qualitative interview study design to identify and understand psychosocial and cultural factors that motivate women to choose and/or participate in unskilled home delivery among postpartum women in Mbarara district, rural southwestern Uganda.

### 2.2. Study Settings

The study was conducted between December 2018 and March 2019 in the Mbarara district of rural southwestern Uganda. Mbarara district is one of the densely populated districts in the Ankole region with one of the highest maternal mortality ratios of 489 per 100,000 women in Uganda [29]. Uganda's public health system is organized into seven tiers with national and regional referral hospitals, general district hospitals, and four levels of community health centers (HC). The village level (HC1) is operated by village health teams (VHTs). The VHTs are community volunteers identified by their community members and are given basic training on major health programs, so they can mobilize and sensitize communities to actively participate in utilizing health services [30]. According to the Uganda Ministry of Health, VHTs also act as an important link between the communities and health facilities and can provide treatment of uncomplicated diseases like malaria, pneumonia, worm infestations, diarrhea, and mass drug administration for Neglected Tropical Diseases. VHTs mobilize communities during specific health campaigns and community disease surveillance activities through active data collection and reporting. Staffing and available services vary across the four levels: HCIII and HCIV should offer Emergency Obstetrics Care (EMOC), whereas HCI and HCII serve as low resource referral units which are not able to provide EMOC and have no ambulances and blood transfusion services [7]. In total, there are about 10 public facilities within a 20 km radius from Mbarara Regional Referral Hospital, the main teaching hospital for the Mbarara University of Science and Technology. Private providers operate in parallel to the public system to provide maternal health care.

### 2.3. Research Team

The research team comprised of seven senior investigators, inclusive of epidemiologists (ECA and CO), an obstetrician (GRM), a medical anthropologist (NCW), a maternal/reproductive health expert (LTM), a nurse (JN), and a health informatics specialist (AM). Based on our previous research and working experiences in maternal health in Uganda, the team sought to explore reasons for low utilization of maternity services in Uganda. The two male and female research assistants were both social scientists, independently hired and trained to conduct research in human subjects. These two research assistants generated transcripts but were not involved in concept development or coding of data. This multidisciplinary team leveraged on their expertise and experience with maternal health issues in Uganda to design, conduct, analyze, and present findings from this study.

### 2.4. Participants and Recruitment

Contact with one of the VHTs based in each of the study villages was initiated by a trained research assistant from the list of VHTs provided by the district health office. A purposive qualitative sampling strategy was used to construct a sample of postpartum women with varied knowledge and experiences of pregnancy, antenatal care, and childbirth. Women who had had their last delivery within the last three months were identified with the help of an existing VHT contact person found in each of the 10 villages located within 20 km from Mbarara Regional Referral Hospital. Trained research assistants initiated a telephone contact to the identified women to seek for permission to visit them for an interview. Research assistants obtained voluntary written informed consent from all eligible participants in the local language in a private area of their homes, communities, or study office. All consenting participants gave written informed consent, or for those who could not write, a thumbprint was made on the consent form. Three women from each of the 10 villages with different home or facility birthing experiences were purposively selected and recruited. The total sample included 15 women who had delivered from their homes and 15 who had delivered from a health facility. Eligible women (1) were adults of childbearing age (18-49 years), (2) had delivered a child in the previous three months, (3) had access to a mobile phone, and (4) were able and willing to give informed consent.

### 2.5. Data Collection

**Figure 1:**
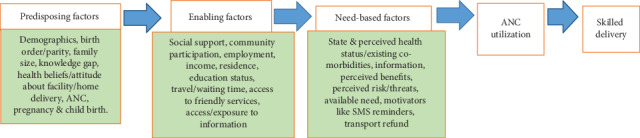
Healthcare service Utilization Model (HUM) adapted to factors relevant to ANC utilization and skilled delivery.

Interview topics were developed using HUM. Data collection consisted of individual, open-ended interviews with each of the study participants (*N* = 30 interviews). A preliminary interview guide was developed and pilot tested by the primary author among women in one of the 10 participating villages using the constructs of the Healthcare service Utilization Model that demonstrates the factors that lead to the use of health services (Figure 1). The guide was revised based on the results of the pilot test. Topics included in the final version of the guide were as follows: (1) perceptions of pregnancy and childbirth, (2) experiences of previous pregnancy or pregnancies, (3) experiences of ANC, (4) engagement with health care providers within a facility, (5) social support, and (6) childbirth experiences. Individualized probes were used to elicit details corresponding to each topic. As the interviews were conducted, emerging content was continuously reviewed by the primary and senior authors to sharpen the interview questions and identify new probes. Demographic information (e.g., age, occupation, and educational background) was collected at the outset of each interview.

All interviews took place in a private location mutually agreed upon by the participant and the interviewer. Interviews were conducted in the local language (Runyankole) and digitally recorded. Interviews lasted 60-90 minutes. Qualitative interviews were digitally recorded with the participant's permission and transcribed. Two Ugandan research assistants transcribed the interviews from the local language directly to English. The two independent male and female research assistants were both social scientists trained in research in human subjects. These two research assistants generated transcripts but were not involved in concept development or coding of data.

### 2.6. Data Analysis and Interpretation

The aim of this qualitative data analysis was to inductively construct categories describing barriers to facility-based delivery. Analysis began with repeated review of transcripts to identify relevant content. The identified content served as the basis for developing a coding scheme. Coding was done in three stages, namely, (1) open coding to identify and describe women's ideas, meaningful expressions, phenomena, or incidents highlighting their experiences during pregnancy and childbirth; (2) axial coding to relate and label codes or data that shared concepts, dimension, and properties (relationship identification); and (3) selective coding to delimit coding to the identified core variables/concepts from the data (Strauss and Corbin, 1998). Data were coded with the aid of the qualitative data management software, NVivo10 (Melbourne, Australia).

Coded data were iteratively reviewed and sorted to identify themes (repeated patterns in the data). Categories were then developed to describe each identified theme. Categories consisted of descriptive labels, elaborating text to define and specify each category's meaning, and illustrative quotes taken from the qualitative data. Data analysis was done jointly by ECA, EA, CO, JN, and GRM. Both JN and ECA coded 5 sampled transcripts and compared the results. Together with GRM and CO, we resolved disagreements until we were satisfied with the consistency in our coding. We aimed to ensure consistency in coding and did not aim for or cite interrater reliability coefficients. Although the interview topics were developed using HUM, we used an inductive content analytic approach to identify and describe psychosocial and cultural factors influencing birthing choices for unskilled home delivery and represented identified influences as descriptive categories.

### 2.7. Ethical Considerations

All consenting participants gave written informed consent, or for those who could not write, a thumbprint was made on the consent form. Permission to conduct the study was obtained from district and local community leaders. The study was reviewed and approved by the Mbarara University of Science and Technology Institutional Ethics Review Committee and the Uganda National Council for Science and Technology, Kampala, Uganda.

## 3. Results

A total of 30 study participants with differing experiences of pregnancy and antenatal care (15 women who delivered from their homes and 15 who delivered from a health facility) were purposively selected from the 10 villages within 20 km from of a referral hospital with the help of existing village health teams and interviewed (Table 1). The median age was 26 years (interquartile range (IQR): 20-33). Twelve women (40%) had completed education beyond primary school. More than half (53%) women reported household income of at least 100,000 Ugandan shillings per month. Most women (60%) reported attending at least two ANC visits and 40% were in formal/legal partnerships. The median time since delivery was 41 (IQR: 23, 65) days. More than half of the referent pregnancies were unplanned. All women reported at least one pregnancy preceding the referent birth, with a median parity of 3 (IQR: 2, 4). Many women in southwestern Uganda (*N* = 9, 30%) would definitely like to deliver from home in the future. Four women (13.3%) delivered from home but would not do it again, while 2 (6.7%) women preferred facility delivery and reported their last home delivery as accidental.

### 3.1. Qualitative Results

The important influencing factors to unskilled home delivery included individual and community sociocultural constructs and/or beliefs about pregnancy and child birth that affected their perceived need for skilled facility maternity care. Women's descriptions of their decision-making processes for facility or home birth revealed that many birthing decisions were largely dependent on previous or current ongoing interpersonal relationships within their social networks. Women's financial dependency on significant others for economic support affects their ability to seek skilled care when they need or want it. The sociocultural birthing expectations as a “natural” and normal nonmedicalized process on the other hand also greatly affects women's perceived need to skilled care especially in women who are poor and financially dependent on significant others for economic support (core category). Women also choose home delivery as a means of controlling their own birth processes, so as to conform to cultural norms and show they are loyal and strong. Other women were dissatisfied with facility-based care, from personal birthing experiences or hearsays from their social networks. Other facilitators to home birth included fatalism regarding birth outcomes; access to alternative sources of birthing help within their communities; perceived as affordable, supportive, and convenient; and the existing gender and traditional norms that limited their ability and freedom to make family or health decisions as women.

### 3.2. Financial Dependency and Expectation for a “*Natural*” and Normal Childbirth

In some Ugandan communities, childbirth is seen as a “*natural*” rather than “medical” or something that needs medical help. Childbirth is also perceived as a nonthreatening normal process unless one got “other” life-threatening illnesses such as malaria, HIV, hypertension, or diabetes along the pregnancy itself that necessitated or needed a hospital visit. This perception of pregnancy and childbirth as “*natural*”, normal, and low risk affects women's perceived need to seek skilled facility delivery, encouraging women to opt for home births. The choice for unskilled home delivery is further accelerated by prior women's uneventful “natural” home births experienced or observed within their communities. These birthing expectations, coupled with women's financial dependency on significant others for all economic provisions, further affect their perceived need, birth preparedness, and ability to seek facility care, as women are too poor to prepare or afford transport and birthing requirements. Women are also unable to mobilize active financial or physical help from their caretakers with other competing demands and/or who see no critical need to go to hospital for such a “*natural*” or “*normal*” birth. Although public and parallel private maternity services are largely available at every subcounty level in Uganda, women's decision to seek professional help was limited in instances where women needed permission and/or financial support from their significant others to access skilled care. An 18-year-old mother of two said,

*I am not the type that gets sickly all the time, apart from common cough and flu. I was never sick throughout my pregnancy and so I didn't need to go to hospital…I think pregnancy is not a disease. Its normal. Its natural. If your baby is kicking properly and you are not sick, you can't be bothering people (caretakers) all the time. You can't just go or ask for money to just go to hospital to waste people's time all the time…we think about children's school fees, food and other things. I go to hospital when I can (afford) and like when I have a problem but I don't always have enough (money) to go or buy stuff so I wait until my husband gives me money or something…if you are not stressing the baby, you just wait for the day of labor and you deliver, life goes on, like that. You have to be strong, whether here or hospital, it can be the same baby. That's what my mother and mother-in-law taught me.*

A 22-year-old mother of two added,

*My husband was not at home to give me transport or money to use there [at the hospital] when labor started so I called my mother who helped deliver me from home…of course I couldn't go like that without his permission so I had to wait…after all, those people [at the hospital] can ignore or abandon you and you end up delivering by yourself without anyone caring to know how you did it if you have no money to give them…I was helped by fellow mothers on the ward to deliver the first time and it was a natural and normal birth for me. I didn't feel sick when I was pregnant this time and I think I didn't need a doctor or anyone to deliver my baby this time around so I stayed at home.*

### 3.3. High Perceived Power to Control Own Birth Processes, so as to Conform to Cultural Norms and Expectations

There is a cultural expectation for women to be strong and courageous, and one of the ways they can show this is by “*taking control*” of their birth process so as to earn respect from their community (friends and family members). The women therefore choose home delivery and look for ways to ensure its success by “*taking charge*” and preparing or seeking control over the birth process to minimize complications when receiving a new family member. Women described a learned sense of control and power to avert birth complications from their experienced family or village birth attendants through the use of tried-and-true, nonbiological practices thought to promote delivery without complications. To make sure the home birth is successful, women engaged in traditional herbal remedies and physical uterine manipulations or carried out certain cultural norms/incantations to “*release the baby*” in case she/he was “*hung/held up there*” as serially experienced in previous births or as observed from other “*successful*” births among fellow local women. Women were therefore initiated to see pregnancy and delivery complications as avoidable and controllable with the help of these “*experienced*” family or village birth attendants within their communities who engaged in similar home births “*without any complications*.” Some women even opted to return to their “*experienced*” parents' homes for delivery. According to a mother of four (39 years),

*The first time, I tried [to go to hospital] when labor started, but I later got to learn from my older sisters-in-law, who all have delivered from home, that our mother-in-law delivers us all, or at least, that is how I found it… I go to her home whenever I am to give birth. Of course I do not want to be seen as a weak wife and one who doesn't respect tradition…all of us have delivered here without complications because she gives us enough [herbal] medicines while pregnant to keep strong and prevent us from bleeding after birth or quickly bring down the baby that is hung up there very fast… She gave me this (herb) to tie around my waist during pregnancy and labor to avoid problems. We are strong women and have pushed all our babies on our own. Besides, she also helps to turn the baby in case they are wrongly positioned…She is very experienced [in delivery] and I have delivered all my 4 children from here [home] normally without any problems.*

A young mother of one (18 years) also added,

*I started labor late in the night. The labor came earlier than we expected and it was late so I prepared to deliver from home. My in-laws would also think I fear giving birth or like I am weak and a coward if I start screaming in the night…I have been taught by my mother and witnessed her deliver normally by herself several times so I learnt everything from her, the herbs to take during pregnancy and during labor to take away labor pains, any problems with the baby and position or clean the baby before being born, the technique to deliver on my knees, cutting the cord, bathing and all…I tried and did everything on my own and by the time he came back, he was surprised. I had my baby in my hands and I was busy doing my chores. I think everyone was surprised but my husband loved it...My friends and in-laws now know I am a very strong woman.*

### 3.4. Dissatisfaction with Facility-Based Care

Dissatisfaction with facility-based care was a reason for choosing home delivery for women in this sample. Perceived quality of institutionalized maternity care was influenced by individual experiences with facility delivery and by what women heard from others in their social networks. Some of the previous experiences that led them to prefer home-based delivery were disengagement, rudeness, and a lack of support from skilled health care personnel, which seemed to affect individual and community's confidence, commitment, and need to utilize available skilled care within their communities. Social networks were made up of friends, peers, family members, and community health workers. These ongoing types of relationships, interactions, and/or advice on pregnancy and child birth within their social networks were influential to women's choices for care and delivery, especially based on their shared past experiences with facility and home care. The network's past birthing experiences also seemed to facilitate deep interpersonal connections that brought up a sense of contentment to model or follow in their role model's footsteps. A 29-year-old mother of three said,

*the nurses are not always there at this facility and the few there can abandon you in labor, abuse you, beat you or something so I feared…I had my second baby last year and my friend is the one who took me there [to the TBA]. She has had all her births there [TBA] and she told me all about her good experiences…We had just moved here last year but the nurses at the health facility were rude and didn't care even if I screamed for help. Well, I found her [TBA] caring the last time I delivered there, even when she did not know me at all, so I had my second delivery there as well...after all my friend was there to take care of me when I gave birth.*

Another 35-year-old mother of four added,

*My family believes that all the first born babies or twins should be born at home to allow the family to privately celebrate their heir, or celebrate certain rituals[like eating or preserving the placenta] and these traditions are despised or not allowed at the hospitals. Those nurses can even abuse, embarrass, slap or chase you from the ward if you are caught doing such. I witnessed it when I escorted my sister to the delivery room…although I do not like it, my family values it a lot…there is a saying in Runyankole that when you go to a community that eat rats, you also have to eat them*, literally meaning, when you live in a certain community, you have to adapt to their norms and culture to live in harmony.

### 3.5. Fatalism regarding Birth Outcomes

A certain religious belief in fatalism is inherent in some Ugandan traditional cultures, despite most women being of Christian faith. Here, women believe it is beyond their control to avert maternal or perinatal death, regardless of where they delivered. An individual's cultural and/or religious belief in fate (fatalism) and divine intervention, mostly at the time of delivery, was therefore seen to influence women's choices for home births. Women who seemed fatalistic about the birth outcomes also identified facility delivery as an alternative option to home birth, costly, inaccessible, inconveniencing, and unable to avert any impending deaths if it was ones day to “*meet one's creator*,” thus expressed as “*not worth their additional effort*.” According to a 35-year-old mother of five,

*Going all that way to hospital? I can. I mean, I did for some of my first two children. Others, I had other family challenges, so I could not go because it was not worth the effort. Anything can happen and may be you find the nurse is not there to help you so you are alone there or no other stuff to do at all to save your life or like you got no money to go to another hospital or like that...But well, anyway, anyone can die anytime, anywhere regardless of where you are, here (home), in hospital, anywhere. If it is your day to meet your creator, you go. If it is not your day, well, God knows. But some women are unlucky so they do not make it.*

A 38-year-old mother of two also said,

*I don't have to pay anything to deliver from my mother's house because she is always there. I have not had any problems for all my deliveries at home. I have been lucky and I always pray to God to intervene for me. If I died or had problems, then that would be my fate that day…Our facility is far away and its not exactly that encouraging as people who go there die a lot…a lot of pregnant women are unlucky or something…I think everyone's day is different and if God planned for yoy to die on such a day, well, no one can stop it. Everyone has their turn and if you are lucky, you give birth without any problems. We are different.*

### 3.6. Access to Alternative Sources of Birthing Options within Women's Communities Perceived as Affordable, Supportive, and Convenient

The existence of alternative (to medical facilities) birthing options within the community seemed to influence women's birthing choices for unskilled home delivery. Traditional birth attendants in Uganda, most of which are without formal training, still conduct many deliveries especially in areas with scarce maternity services or financially challenged couples. These alternatives were presented in a positive light as affordable, close to home, easily/readily accessible, convenient, less demanding, and supportive. These alternatives allow women to stay at or close to home while giving birth, enabling them to continue to meet their responsibilities at home especially in instances where these expectant mothers had no one to delegate duties or outsource someone to stay home or with her other children while she went away to seek maternity care services. Once pregnant women interacted with their peers, friends, or family regarding pregnancy and birthing, the availability of these seemingly attractive delivery options for home birth within their communities seemed to actively shape decisions to deliver from home as their first choice, and facility delivery as an alternative in case “*something out of the usual*” happened. This home birth as a preferred or first choice of delivery often affected resource mobilization as these local birth attendants conducted deliveries for free, at a small cost, with some accepting to be paid their small fee later in manageable installments. The unpleasant past interface with the health facility, challenging referral conditions, worsened by a lack of public transport also seemed to encourage people to seek these alternative options within their communities. The existing competing demands, the routine facility requirement for a caretaker to escort and look after the laboring mother, and proximity to alternative care further facilitated women's motivation/intention, based on the learned notion that in fact many births had occurred within their communities without complications. A 27-year-old mother of 3 said,

*My friend has always helped to deliver women at her home. She is always here for me whenever I deliver so, I decided to stay home and deliver all my last two children here [at her home]. My friend stays nearby so I just walk there and she helps me to deliver well in her home for free. She is an understanding lady and others chose to pay her later and that is fine. She charges a small fee for her service…That way, women do not need to prepare much and it is convenient here so I don't have to abandon my children to go away to hospital for days.*

A 30-year-old mother of 4 added

*She [TBA] charges very little for gloves and a mackintosh. She stays close by and so I can just walk there to deliver anytime without anything in case I need to and without leaving my other children alone…I charges very little and I can pay her later other than going away and my children get attacked, sleep hungry or something. It is very convenient…I stay far away [from the hospital] and I cannot just go for days away from my children unless I get something out of the usual [complication].*

### 3.7. Limited Ability and Freedom to Make Family or Health Decisions and Choices as a Woman

Prevailing cultural norms define women as subordinate to men and to the elders in the husband's family. Traditionally, women are responsible for household tasks and child care, and being married (with children) is her key to having land, shelter, and access to her children. Women are also expected to follow the instructions and preferences of their husband and his family. This subordinate status limits women's autonomy to be self-determining when making decisions about birthing and/or health. Our data indeed showed that these existing gender and traditional norms in rural southwestern Uganda limited women's ability and freedom to make their own choices and decisions to seek skilled maternity care. These cultural expectations also seemed to affect effective engagement in discussions on sociocultural and economic challenges, internalization of benefits, and use of facility delivery. Women described their inability to make decisions to seek facility delivery, especially when they were not empowered to make such joint or independent decisions about their own health and well-being. Some women seemed to want home births, but their partners were always not in support of their choices, insisting on them delivering from the facilities and vice versa. Data from our study further revealed that whereas many women who had had a facility birth felt comfortable recommending a facility birth to someone, some women said they intended to give birth at home in the future as a personal decision or as expected from their families. According to a 22-year-old mother of three,

*we [women] can't talk anyhow, like that to these people [husbands and their relatives] or like ask to go with you [to hospital] all the time...People in this village or his relatives will think you are the man in the house, giving instructions for your husband to follow or like their son is bewitched or weak or something…He's also not easy and I sometimes fear telling him about what the nurses taught us at the hospital, so I keep quiet until he asks me…even if I prefer delivering at home at my friend's house, I know he will never allow me so I have to go to hospital and get ready.*

A 36-year-old mother of four also said,

*“We lost our first born two weeks after delivering at home. He just started breathing badly and by the time we arrived at the hospital, he was gone. There was nothing the nurses could do…I still believe if we had gone earlier, may be, just maybe he would have survived. But unfortunately I have to follow the traditions I found here, like I can't go anywhere without my husband or mother-in-law's permission”.*

Another 31-year-old mother of 2 added,

*I am a woman and I am expected to do certain things and take care of everyone at home, whether pregnant or not…I have to do as my husband pleases because he married me, like if he says I deliver at her mother's house, that's what I have to do to avoid problems with her. I was taught by my mother and others that that is my role [to respect my husband's wishes] and everyone expects that from me as someone's wife.*

## 4. Discussion

The psychosocial and cultural understanding of pregnancy and childbirth is crucial in influencing birthing choices for unskilled home delivery among postpartum women in rural southwestern Uganda. Across both the facility and home delivery groups, our results indicate that there still exists a strong cultural pull for women to choose home births. This paper shows the idea that women choose home delivery (1) because of financial dependency and expectation for a “*natural*” and normal childbirth, affecting their ability and need to seek skilled facility delivery; (2) as a means of controlling their own birth processes, so as to conform to cultural norms and show they are loyal and strong; (3) dissatisfaction with facility-based care from personal birthing experiences or hearsays from their social networks; (4) fatalism regarding birth outcomes; (5) access to alternative sources of birthing help within the community, perceived as affordable, supportive, and convenient; and (6) the existing gender and traditional norms that limit their ability and freedom to make family or health decisions and choices as women. Given the importance of ethnic sociocultural beliefs, perceptions, values, and misconceptions about pregnancy and childbirth in shaping birthing choices and health utilization among specific communities, this study identifies crucial areas that could be acknowledged in designing patient-centered and context-specific health solutions to educate, empower, and motivate these different women to overcome such barriers to care and utilize available skilled maternity care services.

Individual psychological or sociocultural perceptions about pregnancy and child birth influence decisions to choose home birth. Our study showed that child birth, child bearing, and birth processes have unique sociocultural meanings that were learned or experienced within their routine cultural practices. These cultural norms and meanings influence a consistent notion and desire of engagement as well as a faith in one's ability to take control of their own birth processes. The sense and desire of self-satisfaction and personal control during child birth have previously been seen to play a key role in women's choices of birth location even in many developed countries such as the United States, where awareness and need for skilled facilities are high [31–33]. Unlike the solitary Ugandan setting where most women deliver at home alone, there is improved access to public transport, ambulance services, or presence of a skilled personnel at the homes in these developed countries. In our study, many women used generational herbs or rituals to “*bring down*,” “*descend the baby*,” that was “*high up*” or avert any intending complications, creating a sense of power and control to overcome impending challenges and deliver normally as expected.

The cultural norms, expectations, and traditions such as the firstborn children, twin births, and mother-in-law traditional birth attendants influence some women to deliver from home, as a sign of loyalty and strength. The notion of fatalistic belief or tendencies from some women to believe that birthing complications and outcomes are beyond human control and predetermined by God was also not surprising, as have been documented in other similar studies elsewhere [34]. Noteworthy, the anxiety from such expectations and pressures have been listed to lead to delay or no referrals and/or solitude without proper support and preparation to access the needed skilled services in time among African women delivering at home [11, 34–36]. With the inevitable delays and anxiety, these “*experienced*” birth attendants managing these women resort to traditional herbs and maneuvers that could cause several complications, including rapture of the uterus that requires Emergency Obstetrics Care that are common and yet not readily available in most rural communities, to avert imminent risk of death.

Pregnancy and childbirth in Uganda commands power, security, and respect and are a source of sociocultural identity and continuity in many communities [3]. Sociocultural stereotypical identity and expectations of women, and their role in affecting communication, participation in major health or economic decision-making, and life outcomes has been previously described [37]. Noteworthy, many women in Uganda are economically and socially vulnerable, depending on their spouses and significant others to fulfil all their individual and children's financial needs. This poverty and financial dependency affect women's decision-making power in family affairs, health care access, and utilization as they are not free to make independent decisions to seek professional help or spend their own income as and when they need to, because of the culture and patriarchal society as noted by other scholars elsewhere such as in Pakistan [38]. In our study, financial dependency affects women's ability to access care whenever needed and affects women to make independent decisions to seek care or mobilize enough resources to adequately prepare for childbirth. This dependency and disempowerment among women further affect women's perceived need to seek skilled facility care, perceived as unnecessary or not a priority especially in poor women who expect labor to begin and progress naturally and normally. Financial dependency and disempowerment also seem to affect communication, interaction, or discussion of health matters and logistics with partners and/or health care providers, which according to some studies [34, 39] underscores the notion of risk as an important result of these meaningful interactions. These sociocultural experiences, financial vulnerability, low risk perception of pregnancy and childbirth, and expectations of natural birth as an ordinary rite of passage, coupled with women's inability to make independent decision, have been reported to shape women's behavior, attitude, and intention to seek professional health care [40–43].

Dissatisfaction with facility-based care can affect health care utilization, especially where users are not confident of the quality of care provided or cost-to-care is presumed high [44–46]. According to previous studies in Uganda, Tanzania, and Guatemala, poor quality care, bad experience with formal facility delivery, or facility health care dissatisfaction influence women's commitment and choice to give birth in health facilities [41, 46–48]. From our study, an individual's perception about the quality of maternity care and their lack of appreciation of their need to deliver in the available health facilities affected facility utilization, especially if women and their partners perceived facility delivery to be unable to offer them better birthing outcomes, mistrusted, or lacked joint or autonomous power to make firm facility birthing decisions. This combination of women's perceived need for facility-based childbirth services, their perceptions of institutional childbirth care, derived from hearsay or second/third hand accounts of others, and their first-hand experiences of institutional childbirth care has been documented to influence women's intentions to give birth in a health facility in the past and future [41, 49–54].

Women who interpret the available alternative sources of birthing help within their communities as cheaper, supportive, acceptable, and convenient choose home delivery especially where flexibility and competing demands outweigh the desire or capacity to overcome challenging referral conditions to access facilities in financially, psychologically, and physically unprepared couples. The availability of these alternative and unregulated birthing options within the community seemed to affect early resource mobilization among couples, which eventually affected women's transportation or referrals in case there was a need to access professional help. Our data also indicated that women's interactions and relationships within their homes and communities influence their choices for unskilled births from these alternative sources of help. Some of these relationships or social networks, however, were seen to help women to overcome some barriers to skilled care such as helping financially to transport or provide for them, making emergency calls to their significant others, offering to stay home with children as they sought care, helping with house chores, or escorting them to birthing locations during the night. Such role of instrumental support from social networks especially whenever the networks perceived their help crucial, valuable, and important or considered their help as part of their responsibility to assist a vulnerable person in need has been previously been documented [55]. On the other hand, social networks have been found to influence decisions to seek care through health information sharing on their perceived healthcare quality, evaluation of previous care, and their willingness to recommend a certain location or facility to others that previously worked for them or their significant others [56–58]. Whereas social networks have been found to play a key role in childbirth location choices, other factors such as convenience, flexibility, proximity, and level of acceptance have been mentioned as some of the other barriers to skilled facility births, heightening women's choices to deliver from home [34, 36, 40, 59–61].

Our study had a number of strengths. The analysis documents beliefs and perceptions about pregnancy and child birth that influence women's choice to deliver from homes in rural southwestern Uganda, derived using inductive content analytic approach [62]. Influences were identified, developed, and presented as descriptive categories on the basis of their salience in the data set and their originality as contributions to the research literature on health care utilization. This analysis can therefore help inform the design of context-specific health information to educate, empower, and motivate women to utilize available maternity care services in similar communities or settings. This data also highlights the crucial need for improved community-health care provider interactions, as an opportunity to identify sociocultural problems and educate communities to debunk misconceptions about pregnancy and childbirth. These interactions can also be an important step to engaging couples in discussing birth or care goals to improve birth preparedness and perceived need to deliver from available facilities.

Our study also had limitations. This study utilized a sample size of 30 women. However, the study yielded a lot of rich informative data on health facility utilization and home births among rural women in southwestern Uganda. Like most qualitative studies, the goal of this study was not to generalize but rather to provide a deeper contextualized understanding of the rationale and choices for home-based versus skilled facility delivery among recent postpartum birth parents in southwestern Uganda.

## 5. Conclusion

Across both the facility and home delivery groups, our results indicate that there still exist numerous economic challenges, as well as strong sociocultural beliefs and expectations that influence women's choices for unskilled home deliveries in rural southwestern Uganda. Women's financial dependency, established traditions, and perceptions of risk, control, need, and quality of available maternity care at a particular birthing locations influence women's decisions to pursue home delivery in the past and the future. Interventions to address barriers to healthcare utilization through a multipronged approach to teach positive health benefits from antenatal care visits, clean delivery, birth preparedness, skilled delivery, avoidance of harmful traditional practices, such as herbs and birth maneuvers, and mobilizing community support to mobilize resources to financially and physically support their women could be a very useful approach. Addressing specific health beliefs about “*power to control*” birthing processes, fatalism, all-time expectation of childbirth as a natural and “*normal*” process, and cultural pressures for women to experience natural or home birth to prove strength, resilience, and loyalty could help to debunk misconceptions, improve perceived need, and motivate women to seek skilled delivery.

## Figures and Tables

**Table 1:** Demographic characteristics of participants.

Characteristic	Study participants (*n* = 30)
Median age (IQR)	26 (20.33)
Partner age (IQR)	31 (24.45)
Religion	
Catholics	9 (30.0)
Protestants	11 (36.7)
Moslems	5 (16.7)
Others	5 (15.7)
Education level, n (%)	
>Primary	12 (40.0)
≤Primary	13 (43.3)
None	05 (16.7)
Median parity (IQR)	3 (2.4)
Able to read English or Runyankole	25 (83.3)
Regular income (yes)	08 (26.7)
Household income ≥ 100,000 UGX/month	16 (53.3)
Married/legal partnership	12 (40)
Known HIV status	20 (66.7)
Most recent pregnancy planned	13 (43.3)
ANC visits	
0	3 (10.0)
1	09 (30.0)
2-3	11 (36.7)
4-5	5 (16.7)
>5	2 (06.7)
Median time since last delivery (IQR)	41 (23.65)
Median number of people providing the participant with any kind of social support (IQR)	10 (5-16)
Choices for delivery	
Would definitely like to deliver from home in the future	9 (30.0)
Delivered from home but would not do it again	4 (13.3)
Preferred facility delivery but reported their last home delivery as accidental	2 (6.7)
Delivered from facility and would like to do so in the future	10 (33.3)
Delivered from facility but unsure if they would go again	3 (10.0)
Delivered from facility but would not do it in the future	2 (6.7)

## Data Availability

The data used to support the findings of this study are available from the corresponding author upon request.
